# Supplementary material for: Conditional chemoconnectomics (cCCTomics) as a strategy for efficient and conditional targeting of chemical transmission
Source: eLife. 2024 Apr 30;12:RP91927. doi: 10.7554/eLife.91927 (PMC11060718; doi:10.7554/eLife.91927)
Supplement: Figure 3—source data 3. [file elife-91927-fig3-data3.zip › Figure 3 source data3 part 1.zip!/Figure 3-Source data 3-part1/1063f3-1-CRISPResso_Report_kboyr13q.zip!/Nucleotide_percentage_quilt_around_sgRNA_AATCACACCAGTGGGAGCAC.pdf]

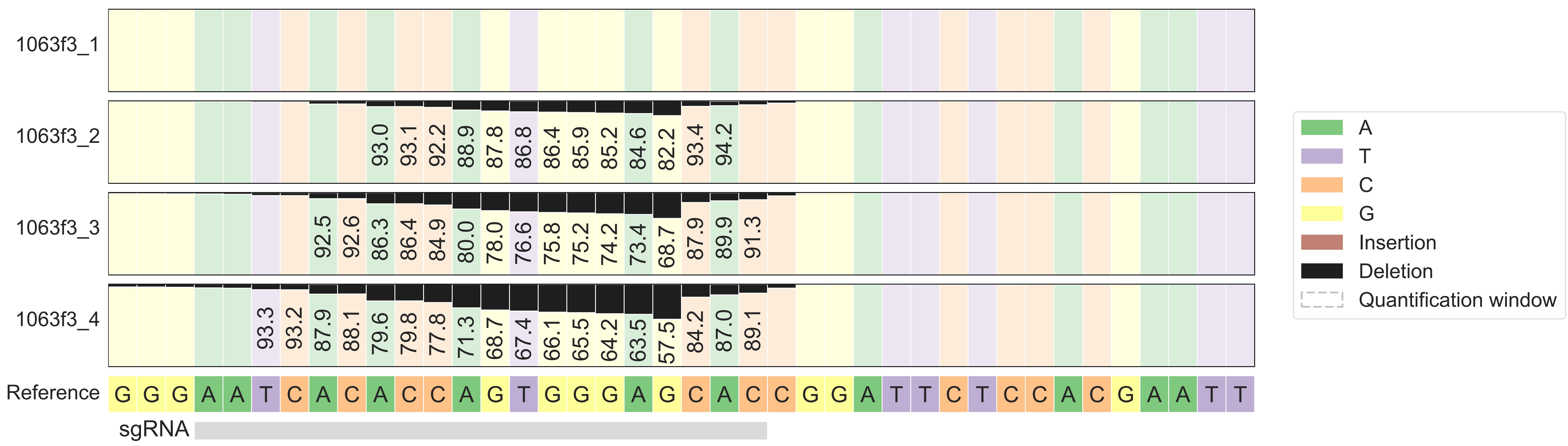

1063f3_1
1063f3_2
93.0 93.1 92.2 88.9 87.8 86.8 86.4 85.9 85.2 84.6 82.2 93.4 94.2
1063f3_3
92.5 92.6 86.3 86.4 84.9 80.0 78.0 76.6 75.8 75.2 74.2 73.4 68.7 87.9 89.9 91.3
1063f3_4
93.3 93.2 87.9 88.1 79.6 79.8 77.8 71.3 68.7 67.4 66.1 65.5 64.2 63.5 57.5 84.2 87.0 89.1
Reference
G G G A A T C A C A C C A G T G G G A G C A C C G G A T T C T C C A C G A A T T
sgRNA
A
T
C
G
Insertion
Deletion
Quantification window
